# Supplementary material for: The causal role of circulating inflammatory markers in osteoporosis: a bidirectional Mendelian randomized study
Source: Front Immunol. 2024 Jul 18;15:1412298. doi: 10.3389/fimmu.2024.1412298 (PMC11291241; doi:10.3389/fimmu.2024.1412298)
Supplement: Supplementary file 1 [file DataSheet_1.zip › supplementary material/OSTEOPOROSIS-CIM/GCST90274801.tsv.gz_forest.pdf]

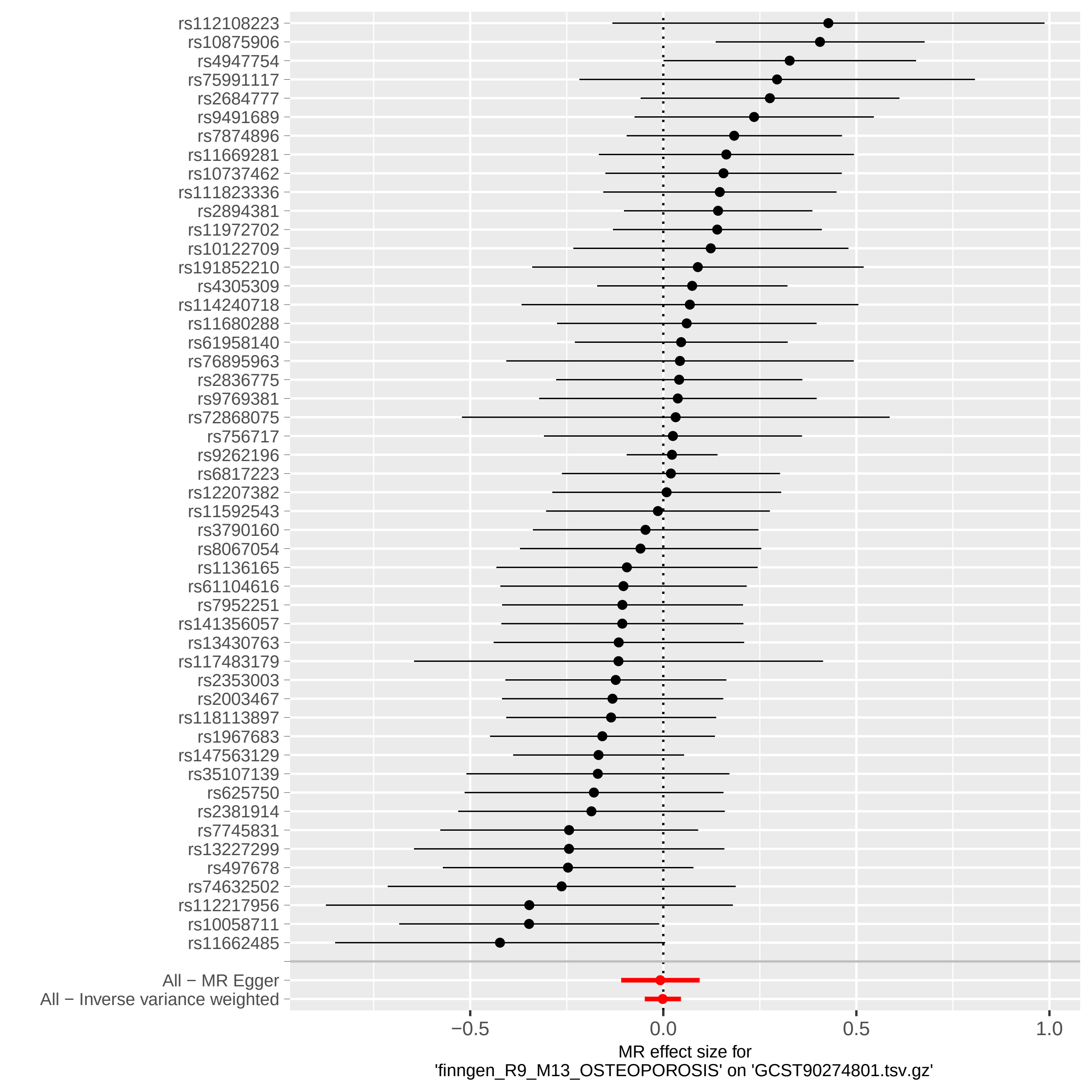

rs112108223
rs10875906
rs4947754
rs75991117
rs2684777
rs9491689
rs7874896
rs11669281
rs10737462
rs111823336
rs2894381
rs11972702
rs10122709
rs191852210
rs4305309
rs114240718
rs11680288
rs61958140
rs76895963
rs2836775
rs9769381
rs72868075
rs756717
rs9262196
rs6817223
rs12207382
rs11592543
rs3790160
rs8067054
rs1136165
rs61104616
rs7952251
rs141356057
rs13430763
rs117483179
rs2353003
rs2003467
rs118113897
rs1967683
rs147563129
rs35107139
rs625750
rs2381914
rs7745831
rs13227299
rs497678
rs74632502
rs112217956
rs10058711
rs11662485
All − MR Egger
All − Inverse variance weighted
−0.5
0.0
0.5
1.0
MR effect size for
'finngen_R9_M13_OSTEOPOROSIS' on 'GCST90274801.tsv.gz'
